# Utility of coronary artery calcium in refining 10-year ASCVD risk prediction using a Thai CV risk score

**DOI:** 10.3389/fcvm.2023.1264640

**Published:** 2023-11-02

**Authors:** Noppanat Tiansuwan, Thinnakrit Sasiprapha, Sutipong Jongjirasiri, Nattawut Unwanatham, Ammarin Thakkinstian, Jiraporn Laothamatas, Thosaphol Limpijankit

**Affiliations:** ^1^ Division of Cardiology, Department of Medicine, Faculty of Medicine Ramathibodi Hospital, Mahidol University, Ratchathewi, Thailand; ^2^ Department of Diagnostic and Therapeutic Radiology, Faculty of Medicine Ramathibodi Hospital, Mahidol University, Ratchathewi, Thailand; ^3^ Department of Clinical Epidemiology and Biostatistics, Faculty of Medicine Ramathibodi Hospital, Mahidol University, Ratchathewi, Thailand; ^4^ Faculty of Heath Science Technology, Chulabhorn Royal Academy, Bangkok, Thailand

**Keywords:** coronary artery calcium (CAC), coronary artery calcium score (CACS), atherosclerotic cardiovascular disease (ASCVD) risk prediction, cardiovascular (CV) risk stratification, Thai CV risk score

## Abstract

**Background:**

Coronary artery calcium (CAC) scanning is a valuable additional tool for calculating the risk of cardiovascular (CV) events. We aimed to determine if a CAC score could improve performance of a Thai CV risk score in prediction of 10-year atherosclerotic cardiovascular disease (ASCVD) risk for asymptomatic patients with CV risk factors.

**Methods:**

This was a retrospective cohort study that enrolled asymptomatic patients with CV risk factors who underwent CAC scans between 2005 and 2013. The patients were classified as low-, intermediate-, or high-risk (<10%, 10%–<20%, and ≥20%, respectively) of having ASCVD within 10-years based on a Thai CV risk score. In each patient, CAC score was considered as a categorical variable (0, 1–99, and ≥100) and natural-log variable to assess the risk of developing CV events (CV death, non-fatal MI, or non-fatal stroke). The C statistic and the net reclassification improvement (NRI) index were applied to assess whether CAC improved ASCVD risk prediction.

**Results:**

A total of 6,964 patients were analyzed (mean age: 59.0 ± 8.4 years; 63.3% women). The majority of patients were classified as low- or intermediate-risk (75.3% and 20.5%, respectively), whereas only 4.2% were classified as high-risk. Nearly half (49.7%) of patients had a CAC score of zero (no calcifications detected), while 32.0% had scores of 1–99, and 18.3% of ≥100. In the low- and intermediate-risk groups, patients with a CAC ≥100 experienced higher rates of CV events, with hazard ratios (95% CI) of 1.95 (1.35, 2.81) and 3.04 (2.26, 4.10), respectively. Incorporation of ln(CAC + 1) into their Thai CV risk scores improved the C statistic from 0.703 (0.68, 0.72) to 0.716 (0.69, 0.74), and resulted in an NRI index of 0.06 (0.02, 0.10). To enhance the performance of the Thai CV risk score, a revision of the CV risk model was performed, incorporating ln(CAC + 1), which further increased the C statistic to 0.771 (0.755, 0.788).

**Conclusion:**

The addition of CAC to traditional risk factors improved CV risk stratification and ASCVD prediction. Whether this adjustment leads to a reduction in CV events and is cost-effective will require further assessment.

## Introduction

Coronary artery calcium (CAC) scanning is a valuable additional tool for calculating the risk of cardiovascular (CV) events beyond traditional risk factors (1, 2). Although a pooled cohort equation, such as those used for the Framingham and American College of Cardiology and American Heart Association (ACC/AHA) risk scores, is useful in predicting atherosclerotic CV disease (ASCVD) risk (3, 4), it doesn't encompass all possible risk factors. These risk factors include racial/ethnic minority membership, family history, obesity, sedentary lifestyle, elevated triglyceride and uric levels, and chronic kidney disease (CKD). Moreover, these scores often overestimate the risk of future CV events and do not enable precise targeting of individual preventive treatment (5). Therefore, there is considerable interest in improving current risk models by incorporating nontraditional risk factors such as arterial stiffness index, high-sensitivity C-reactive protein level (hs-CRP), and CAC with the ultimate goal of reducing CV events and mortality (6–8).

Several studies have been published to enhance the accuracy of predicting future CV events using CAC (9–11). It enables a direct visualization of the extent of atherosclerotic burden in coronary arteries which is the underlying cause of most CV events (3). Evidence from the Multi-Ethnic Study of Atheroslcerosis (MESA) suggests that a CAC score of 100 or greater can be a valuable tool for refining ASCVD risk calculation in these populations (12), helping to identifying individuals at higher risk of CV disease yet are classified as low- or intermediate-risk based on traditional risk factors alone. Additionally, CAC has proven useful in guiding the initiation of statin and antiplatelet therapies (12, 13).

It is important to note that ethnic groups vary in their rates of ASCVD, influenced by genetics, environmental, and lifestyle factors. While some ASCVD risk calculators take ethnicity into account (5, 9), the Asian population is still considered a minority. As a result, a Thai CV risk score has been developed and is used to assess the risk of 10-year ASCVD events in the population (14). This score is based on a longitudinal CV cohort study of a Thai population, known as the Electricity Generating Authority of Thailand (EGAT) study (15). Similar to Western risk scores, the incidence of the CV events is higher than expected in the low-risk group and lower than expected in the moderate-to-high risk groups (16–18). Because of inherent uncertainty in prediction, incorporating the CAC may help to develop a more accurate reclassification of the existing risk prediction model. Though CAC has been extensively studied in population-based assessments in the United States and Europe, only a limited number of studies that have utilized CAC to modify ASCVD risk prediction in Asian populations (19, 20). As such, this study was conducted to determine if the scores from CAC scanning could improve performance of a Thai CV risk score in prediction of 10-year ASCVD outcomes in adults who are asymptomatic with CV risk factors.

## Materials and methods

This retrospective cohort study enrolled asymptomatic adults with CV risk factors who underwent CAC scans for the assessment of coronary artery disease (CAD) at the Advanced Diagnostic Imaging Center, Ramathibodi Hospital, Mahidol University, between November 2005 and November 2013. The study protocol was approved by the Ethics Committee of the Faculty of Medicine Ramathibodi Hospital, Mahidol University (# COA.MURA2022/732). Written informed consent was obtained from each participant before performance of the coronary CT study.

Inclusion criteria for study subjects were: (1) age >18 years, and (2) asymptomatic with CV risk factors (19). Exclusion criteria were: (1) history of prior ASCVD [such as myocardial infarction (MI), stroke, or revascularization], (2) high serum creatinine (>1.5 mg/dl), (3) severe asthma, and (4) severe allergy to seafood or contrast agents.

### Data collection

The recorded subject data included age, sex, risk factors [such as smoking, diabetes mellitus (DM), hypertension, and dyslipidemia], body mass index (BMI, kg/m^2^), family history of CAD, and both prior and current treatments. Recorded lab data consisted of fasting plasma glucose (FPG), lipid profile (triglyceride, total cholesterol, low-density lipoprotein cholesterol [LDL-C], high-density lipoprotein cholesterol [HDL-C]), and serum creatinine. DM was defined as an overnight FPG level of ≥126 mg/dl or taking antidiabetic medications. Hypertension was defined as systolic BP (SBP) ≥140 mmHg or diastolic BP (DBP) ≥90 mmHg, or taking anti-hypertensive medications. Dyslipidemia was defined as a total cholesterol ≥200 mg/dl or LDL-C ≥130 mg/dl, or taking a statin medication. Smoking was classified as current smoking, ex-smoking more than 1 month, or never smoked. CKD was defined as an estimated glomerular filtration rate (eGFR) <60 ml/min/1.73 m^2^, calculated using the CKD Epidemiology Collaboration (CKD-EPI) equations (20). Hyperuricemia was defined as a uric acid level ≥7 mg/dl.

As part of the protocol, all patients underwent measurement of arterial stiffness using the cardio-ankle vascular index (CAVI) on the same day as the coronary CT study. This measurement was performed using a Vasera VS-1,000 vascular screening system (Fukuda Denshi, Japan), that was used to measure ankle-brachial index for diagnosis of peripheral arterial disease. The details regarding how CAVI was measured have been published in a previous report (21). In this study, the mean value of right and left CAVIs was used for analyses. According to the manufacturer, values <8.0 are considered as normal, 8.0–<9.0 as borderline, and ≥9.0 as high, suggestive of the presence of arteriosclerosis and predictive of CV risk. In our analyses, we used a cut-off of CAVI ≥9.0, which is generally considered indicative of significant arterial stiffness and is widely utilized for predicting CV risk (22, 23).

### A Thai CV risk score classification

A Thai CV risk score was developed based on the EGAT data (15) to estimate the 10-year absolute CV risk for each individual, considering several variables, such as age, sex, smoking status, DM, SBP, and levels of LDL-C, and HDL-C (15). An online calculator is available at https://med.mahidol.ac.th/cardio_vascular_risk/thai_cv_risk_score. The details of the model variables, the full score, and the predicted probability of an event were in the Supplementary Figure S1. The CV risk score was classified as low-risk (<10%), intermediate-risk (10%–<20%), and high-risk (≥20%).

### Coronary artery calcium scoring

The quantification of the CAC was performed using multidetector coronary CT scanners: a 64-slice scanner (Somaton Sensation 64 eco, Siemens) and a 320-slice scanner (Aquilion ONE, Toshiba) were utilized before and after 2008, respectively. Before transitioning to the new scanner machines, we took several measures to mitigate potential biases and discrepancies between these two scanner types. First, we conducted scanner matching and demonstrated that there were no significant differences in the CAC scores obtained from the two machines. This process included calibrations to ensure that the scores were directly comparable. Second, we implemented scan protocol standardization, ensuring that the same techniques were applied. This encompassed maintaining consistent slice thickness (3-mm), overlap (20%–30%), and utilizing identical acquisition scan parameters. Therefore, quality control of the CAC score was maintained between the two scanner machines.

The Agatston method (24), implemented with a commercially available external workstation (Vitrea fx 3.0.1, Vital Images), was employed to calculate the CAC score. Interpretation of the results was conducted by a Board-certified radiologist. The total CAC score was calculated as the summation of individual lesion scores in all coronary arteries, and was considered both as a categorical variable (0, 1–99, and ≥100) and a continuous variable. For the later, total CAC + 1 score was transformed to natual logarithm because of its skewed distribution.

### Long-term clinical outcomes

Patients were retrospectively followed up for clinical outcomes until the year 2019, and utilized data from four sources: (1) the Information Technology (IT) Department of Ramathibodi Hospital's electronic medical records, (2) the Strategy and Planning Division of the Office of the Permanent Secretary of the Ministry of Public Health (MoPH), (3) the Information and Communication Technology (ICT) Center of the MoPH, and (4) the Center Office for Healthcare Information of the Health Systems Research Institute of the MoPH. The International Classification of Diseases, Tenth Revision (ICD-10) codes were carefully reviewed to best align with the relevant outcomes of interest of each subject.

The composite outcome of interest was major CV events (MACEs) encompassed of CV death, non-fatal MI, and non-fatal stroke. In the case that multiple CV events were experienced by a patient, only the first event was counted. CV deaths were confirmed by death certificates issued by the National Statistics Office of the Ministry of Interior. A myocardial infarction (MI) was defined as an elevation in cardiac troponin (cTn) levels along with one of the following criteria: (1) prolonged ischemia as indicated by chest pain lasting more than 20 min; (2) ischemic ST-segment changes or the presence of new pathological Q waves; (3) angiographic evidence of coronary occlusion or no-reflow/slow flow; (4) imaging showing new loss of viable myocardium or new regional wall motion abnormalities. “Stroke” was defined as a history of ischemic or of hemorrhagic stroke, or a transient ischemic attack (TIA), documented through computerized tomography or magnetic resonance imaging.

### Statistical analysis

Continuous variables (such as age, height, weight, and laboratory data) were described using mean or median where appropriate while categorical variables (such as sex, smoking history and medication use) were described using frequency or percentage. Data were compared between groups using Student's *t*-test (or the Wilcoxon–Mann–Whitney test) and the Chi-squared (or Fisher's exact) tests. A Cox proportional hazard (CPH) model was applied to estimate risk of MACE by fitting the Thai CV risk score with and without ln(CAC + 1) score, adjusting for potential confounding variables such as age, sex, smoking, and other underlying diseases (i.e., DM, hypertension, dyslipidemia, CKD, hyperuricemia, CAVI). The Akaike Information Criteria (AIC) (25) and Baysian Information Criteria (BIC) (26) were applied to select if ln(CAC + 1) score or categorical CAC score was better fitting. The performance of the CPH models, containing only Thai CV risk score or with CAC variables, were assessed and compared using Harrell's C statistic index and net reclassification improvement (NRI) index. All analyses were performed using STATA 17.0. (Stata Statistical Software: Release 17; StataCorp). A *p*-value of less than 0.05 was considered statistically significant.

## Results

A total of 6,964 asymptomatic patients with CV risk factors were enrolled for analysis. The patients had a mean (± SD) age and BMI of 59.0 ± 8.4 years and 24.9 ± 3.6 kg/m^2^, respectively, with 63.9% being female. ASCVD risk factors were common with 64.1% having hypertension, 61.1% dyslipidemia, 25.7% DM, 6.2% CKD, and 14.7% being current/ex-smokers.

The majority of patients were classified as low- or intermediate-risk (75.3% and 20.5%, respectively), with the remaining 4.2% as high-risk. The distribution of CAC scores were as follows: 49.7% had a CAC score of zero, 32.0% had a score of 1–99, and 18.3% had a score of ≥100. The patient's risk factors (from Thai CV risk classification) and CAC scores were compared (Supplementary Table S1). As expected, there was a positive association between Thai CV risk classifications and CAC scores. The low-risk group had the highest percentage of CAC scores of zero (57.9%), followed by the intermediate-risk group (25.9%), and the high-risk group (17.4%). On the other hand, the high-risk group had the highest percentage of CAC score ≥100 (50.7%), followed by the intermediate-risk group (35.5%), and the low-risk group (11.8%). Futhermore, the median ln (CAC + 1) was highest in the high-risk group [4.7 (range: 0, 8.5)], followed by the intermediate-risk group [3.7 (range: 0, 8.1)], and lowest in the low-risk group [0 (range: 0, 8.2)].

### Association of risk factors, CAC and long-term CV events

The 6,964 patients were followed for 66,696 person-years, with the average follow-up period being 9.9 ± 2.4 years. During this period, 562 MACEs occurred, comprised of CV death (8.5%), non-fatal MI (28%), and non-fatal stroke (75.5%). The study found that patients with a CAC scores of 1–99 and ≥100 had MACE rates of 8.29 and 18.39 per 1,000 person-years, respectively, whereas the patients with a CAC score of zero had a rate of 5.30, with the corresponding hazard ratios (HRs) (95% CI) being 1.59 (1.29, 1.95) and 3.61 (2.96, 4.42) (Table 1). Additionally, there was a positive correlation between ln (CAC + 1) and MACE, with an HR of 1.24 (1.20, 1.29).

**Table 1:** Factors associated with major CV events (MACEs).

Factor	MACEs (%) *n* = 562	Time at risk (year)	Incident rate 1,000 persons/year	Hazard ratio (HR)	95% CI	*P*-value
Thai CV risk score						
Low <10%	5.40	51,020.01	5.55	1.00		
Intermediate 10%–<20%	14.09	13,083.01	15.36	2.81	(2.34, 3.36)	<0.001
High ≥20%	26.53	2,593.20	30.08	5.51	(4.28, 7.07)	<0.001
CAC score level						
CAC 0	5.26	34,366.21	5.30	1.00		
CAC 1–99	7.89	21,237.00	8.29	1.59	(1.29, 1.95)	<0.001
CAC ≥100	16.00	11,092.99	18.39	3.61	(2.96, 4.42)	<0.001
CAVI						
≥9	12.33	24,412.56	15.44	1.97	(1.69, 2.29)	<0.001
<9	5.57	40,510.42	7.68	1.00		
Gender						
Male	9.86	24,030.13	10.32	1.39	(1.18, 1.65)	<0.001
Female	7.06	42,666.08	7.36	1.00		
Hypertension						
Yes	10.87	42,059.27	11.53	3.73	(2.93, 4.74)	<0.001
No	3.08	24,618.03	3.13	1.00		
DM						
Yes	13.62	16,706.45	14.61	2.32	(1.96, 2.74)	<0.001
No	6.15	49,989.76	6.36	1.00		
Dyslipidemia						
Yes	11.30	39,785.22	12.09	4.13	(3.265, 5.23)	<0.001
No	3.00	26,892.08	3.01	1.00		
Current/ex-Smoking						
Yes	10.57	9,738.13	11.09	1.38	(1.12, 1.71)	0.002
No	7.64	56,958.08	7.97	1.00		
CKD						
Yes	17.44	3,703.93	20.25	2.74	(2.14, 3.49)	<0.001
No	7.45	62,982.12	7.73	1.00		
Uric acid ≥7 mg/dl						
Yes	12.49	8,432.08	16.37	1.67	(1.39, 2.02)	<0.001
No	7.43	56,902.41	9.79	1.00		
Statins						
Yes	10.52	44,857.83	11.15	4.01	(3.082, 5.225)	<0.001
No	2.80	21,838.38	2.84	1.00		
Family history of CAD						
Yes	7.12	22,463.43	7.43	0.83	(0.69, 0.997)	0.046
No	8.56	44,232.79	8.93	1.00		
				HR	95% CI	*P*-value
Age (year), mean ± SD	63.77 ± 8.38			1.08	(1.07, 1.09)	<0.001
BMI, kg/m^2^, mean ± SD	25.21 ± 3.75			1.03	(1.00, 1.05)	0.018
SBP (mmHg), mean ± SD	137.94 ± 19.83			1.02	(1.01, 1.024)	<0.001
TC (mg/dl), mean ± SD	200.87 ± 41.62			0.996	(0.993, 0.998)	<0.001
HDL-C (mg/dl), mean ± SD	50.88 ± 13.53			0.994	(0.988, 1.000)	0.058
LDL-C (mg/dl), mean ± SD	125.45 ± 38.56			0.995	(0.992, 0.997)	<0.001
Ln (CAC + 1), median (range)	3.4 (0.0, 8.0)			1.24	(1.20, 1.29)	<0.001

BMI, body mass index; CAC, coronary artery calcium; CAD, coronary artery disease; CAVI, cardio-ankle vascular index; CKD, chronic kidney disease (eGFR <60 ml/min/1.73 ml^2^); DM, diabetes mellitus; HDL-C, high-density lipoprotein cholesterol; LDL-C, low density lipoprotein cholesterol; SBP, systolic blood pressure; TC, total cholesterol.

Patients with high-, intermediate- and low-Thai CV risk scores also exhibited MACE rates of 30.08, 15.36, and 5.55 per 1,000 person-years with HRs of 5.5 (4.28, 7.07), and 2.81 (2.34, 3.36), respectively. MACE occurrences were also associated with traditional risk factors [old age, BMI, male gender, hypertension, DM, dyslipidemia, current or ex-smoking, CKD, hyperuricemia, and arterial stiffness index (CAVI ≥9)].

### Effect of additional CAC and Thai CV risk classification on MACEs

The study examined the effect of CAC score, in combination with a 10-year Thai CV risk classification, on prediction of MACEs (Table 2). The findings revealed that within the low-CV risk group, patients with a CAC score ≥100 and 1–99 exhibited higher incidences of MACEs than did those with a CAC score of zero (11.91, 6.15 and 4.08 per 1,000 person-years, respectively), and HRs of 3.04 (2.26, 4.10) and 1.54 (1.18, 2.02). In the intermediate-CV risk group, only patients with a CAC score ≥100 demonstrated a higher incidence of MACEs (per 1,000 person-years) compared to those with a CAC score of zero, or 1–99, resulting in an HR of 1.95 (1.35, 2.81). Importantly, in the high-CV risk group, the magnitude of CAC score provided no additional accuracy of the prediction of future MACEs.

**Table 2:** Incidence of MACEs grouped by risk category and CAC score.

Factor	Time at risk (year)	No. subject	No. event	incident rate 1,000 persons/year	Hazard Ratio	95% CI	*P*-value
Low-risk							
CAC ≥100	5,540.78	620	66	11.91	3.04	(2.26, 4.10)	<0.001
CAC 1–99	15,114.65	1,586	93	6.15	1.54	(1.18, 2.02)	0.002
CAC 0	30,364.57	3,037	124	4.08			
Ln(CAC + 1)					1.20	(1.14, 1.26)	<0.001
Intermediate-risk							
CAC ≥100	4,291.23	506	96	22.37	1.95	(1.35, 2.81)	<0.001
CAC 1–99	5,262.20	551	64	12.16	1.03	(0.70, 1.53)	0.865
CAC 0	3,529.57	370	41	11.62			
Ln(CAC + 1)					1.13	(1.06, 1.20)	<0.001
High-risk							
CAC ≥100	1,260.98	149	42	33.31	0.99	(0.56, 1.74)	0.96
CAC 1–99	860.15	94	19	22.09	0.62	(0.32, 1.20)	0.158
CAC 0	472.08	51	17	36.01			
Ln(CAC + 1)					1.03	(0.94, 1.14)	0.491

The risk of MACEs was further assessed in those patients with CAC scores of zero. Subjects were divided into three groups based on their CV risk scores. The unadjusted HRs (95% CI) for intermediate-risk and high-risk groups compared to the low-risk group were 2.89 (2.03, 4.12) and 8.89 (5.36, 14.78), respectively.

The models that included ln(CAC + 1) yielded better fitting than categorical CAC groups, with higher AIC in the former than the later (Supplementary Table S2). Thus, ln(CAC + 1) was included in the rest of the analyses. Harrell's C statistics were estimated and demonstrated that the model which contained only the Thai CV risk score had Harrell's C statistic of 0.703 (0.68, 0.72), while incorporation of ln(CAC + 1) into this model improved the Harrell's C statistic to 0.716 (0.69, 0.74) (Table 3).

**Table 3:** Comparison of model performance between those containing the Thai CV risk score with vs. without addition of ln(CAC + 1).

	Hazard ratio	95% CI	*P*-value	Harrell's C statistic	95% CI
Thai CV risk score	1.96	(1.81, 2.12)	<0.001	0.703	(0.68, 0.72)
Thai CV risk score	1.76	(1.62, 1.92)	<0.001	0.716	(0.69, 0.74)
Ln(CAC + 1)	1.12	(1.07, 1.16)	<0.001		

#### Net reclassification improvement (NRI)

To assess the impact of the CAC scores on ASCVD risk reclassification, we computed the NRI between the two models [Thai CV risk alone in row, and Thai CV risk plus ln(CAC + 1) in column; Table 4]. Of the 562 MACEs, 52 (9.2%) and 28 (5.0%) patients who were initially assessed as low- and intermediate-risk using the Thai CV risk model were reclassified after inclusion of CAC scores as intermediate- and high-risk, respectively. However, 24 (4.3%) and 16 (2.8%) patients classified initially as intermediate- and high-risks were incorrectly reclassified when using the model which included CAC scores as low-risk and intermediate-risk, respectively.

**Table 4:** Net reclassification improvement (NRI) index comparing the model with Thai CV risk and Thai CV risk plus ln(CAC + 1).

Thai CV risk score	Thai CV risk score + ln(CAC + 1)			Total (%)
	Low-risk (*n*, %)	Intermediate-risk (*n*, %)	High-risk (*n*, %)	
Up-reclassification for MACEs				
Low-risk	231 (41.1)	52 (9.2)	0	283 (50.4)
Intermediate-risk	24 (4.3)	149 (26.5)	28 (5.0)	201 (35.7)
High-risk	0	16 (2.8)	62 (11.0)	78 (13.9)
Total (%)	255 (45.4)	217 (38.6)	90 (16.0)	562
Down-reclassification for non-MACEs				
Low-risk	4,638 (72.5)	322 (5.0)	0	4,960 (77.5)
Intermediate-risk	285 (4.4)	846 (13.2)	95 (1.5)	1,226 (19.1)
High-risk	0	56 (0.9)	160 (2.5)	216 (3.4)
Total (%)	4,923 (76.9)	1,224 (19.1)	255 (4.0)	6,402

Total NRI = 0.06 (95% CI 0.02, 0.10), *P*-value 0.001.

Among 6,402 subjects without MACEs, 285 (4.4%) and 56 (0.9%) were downgraded when using the model which included CAC scores from intermediate-risk to low-risk, and high-risk to intermediate-risk, whereas 322 (5.0%) and 95 (1.5%) were incorrectly upgraded from low-risk to intermediate-risk, and intermediate-risk to high-risk, respectively. As a result, the overall NRI (95% CI) was 0.06 (0.02, 0.10), indicating that adding ln(CAC + 1) into the Thai CV risk calculation could significantly improve prediction of MACEs 6%.

### Revision of CV risk model

To improve the performance of the Thai CV risk score, we revised the prediction model so that ln(CAC + 1) was considered along with both other risk factors (age, gender, smoking, DM, CKD, hyperuricemia, hypertension and dyslipidemia) and arterial stiffness index (CAVI ≥9). In the revised model, the latter two comorbidities (hypertension and dyslipidemia) replaced SBP and lipid profiles (LDL and HDL) in the Thai CV risk calculation because they accounted for the potential impact of concurrent treatments. As a result, the group of risk predictors were associated with more significant HRs for the development of MACEs. This revised set of risk factors were: age, male gender, hypertension, DM, dyslipidemia, hyperuricemia, CAVI, and ln(CAC + 1) (Table 5). After adjusting for covariates, every one-unit of ln(CAC + 1) was associated with an increased MACE risk, with a HR of 1.16 (1.12, 1.20). This revised model further increased the discriminative Harrell's C statistic (95% CI) from 0.716 (0.69, 0.74) to 0.771 (0.755, 0.788).

**Table 5:** Risk predictors including ln(CAC + 1) and associated hazard ratios for the development of MACEs using the revised prediction model.

	Hazard ratio (95% CI)	Std. Err.	Beta coefficient (95% CI)	Std. Err.	*P*-value
Ln(CAC +1)	1.16 (1.12–1.20)	0.02	0.15 (0.11–0.18)	0.02	<0.001
Age, years	1.03 (1.02–1.04)	0.01	0.03 (0.02–0.04)	0.01	<0.001
Male	1.37 (1.15–1.64)	0.13	0.32 (0.14–0.50)	0.09	0.001
Hypertension	1.70 (1.34–2.16)	0.21	0.53 (0.29–0.77)	0.12	<0.001
DM	1.40 (1.20–1.64)	0.11	0.34 (0.18–0.49)	0.08	<0.001
Dyslipidemia	3.21 (2.56–4.03)	0.37	1.17 (0.94–1.39)	0.12	<0.001
Smoking	1.11 (0.90–1.37)	0.12	0.11 (−0.11,0.32)	0.11	0.328
CKD	1.14 (0.89–1.46)	0.14	0.13 (−0.11–0.38)	0.13	0.284
Uric acid ≥7 mg/dl	1.26 (1.03–1.54)	0.13	0.23 (0.03–0.43)	0.10	0.025
CAVI ≥9	1.20 (1.02–1.41)	0.10	0.18 (0.02–0.35)	0.08	0.030

Abbreviations as in Table 1.

CKD (eGFR <60 ml/min/1.73 m^2^); DM (overnight fasting plasma glucose level of ≥126 mg/dl or taking antidiabetic medications); Dyslipidemia (total cholesterol ≥200 mg/dl or LDL-C ≥130 mg/dl or taking a statin medication); Hypertension (systolic BP ≥140 mmHg or diastolic BP ≥90 mmHg or taking anti-hypertensive medications).

Harrell's C statistics (95% CI) = 0.771 (0.755, 0.788).

## Discussion

In the present study, we demonstrated that incorporating CAC scoring into the Thai CV risk score leads to improved ASCVD prediction with Harrell's C statistics from 0.706 to 0.713. In addition, inclusion of CAC scores improved ASCVD prediction with a NRI index of 6%. Revision of the CV risk prediction model to consider ln(CAC + 1), arterial stiffness index (CAVI ≥9), hyperuricemia, and other CV risk factors (specifically, considering hypertension and dyslipidemia as comorbidities rather than measures of SBP and lipid profile) further improved the Harrell's C statistic to 0.771.

Regarding the prediction of future CV events, both categorical and continuous CAC scores were associated with MACEs, but their effect was less than the Thai CV risk score. This observation served as a reminder that CAC alone is merely a surrogate marker of coronary plaque burden and represents one of several additional risk predictors that should be considered alongside other clinical risk factors.

There is no universally agreed-upon cut-off for CAC scores to modify MACE prediction beyond the ASCVD risk scores. Several researches (12, 23) suggest a cut-off of CAC score ≥100 be used to reclassify an individual' risk category. As noted, the majority (over 95%) of our study cohort were classified as having low to intermediate ASCVD risk, and only 20% of patients had a CAC score ≥100. Among those in the intermediate-risk group, patients with CAC ≥100 experienced a higher rate of MACEs, while those in the low-risk group with either a CAC score of >1–99 or ≥100 had elevated rates. In our study, besides the intermediate-risk group, which would benefit the most and is suitable for using CAC to reclassify risk, we found that the low-risk group could also benefit from use of CAC. This was contradictory to the recommendation from the Society of Cardiovascular Computed Tomography (SCCT) (1, 27) that use of CAC scanning be primarily for intermediate-risk subjects (with a 10-year ASCVD risk of 5%–20%). Several explanations may account for our finding: (1) differences between the classification of the Thai CV risk score and other ASCVD risk scores (in our study, the low-risk group represented <10% of risk rather than <5%); (2) unidentified risk factors, such as genetic or family history of CAD and smoking status, were not factored into our risk score (these factors can impact future CV events, even in patients classified as low risk); (3) ethnic differences in the frequency and severity of coronary atherosclerosis (28, 29). Further studies are needed to better understand the optimal role of CAC scanning in low-risk individuals with a family history of premature CAD (27, 30), cigarette smoking (31), or metabolic syndrome (32, 33). Importantly, our results were aligned with other recommendations from the SCCT (27), indicating that CAC scores do not provide additional predictive benefit in patients already having high clinical risk scores (>20%). For these patients, management should focus on addressing identified risk factors, implementing lifestyle changes, and prescribing statin or aspirin treatment, without the need for CAC scanning.

Almost half of our patients had a CAC score of zero. There is some uncertainty about the power of a CAC score of zero and whether it can be used as a tool to guide patients toward discontinuing preventive treatement. Many previous studies (34–38) have shown very low CV event rates among those with a CAC score of zero, suggesting that this score indicates a low 10-year risk and could be used to downgrade a patient's risk classification. However, our results showed some differences from these earlier reports which were based on Western populations. A CAC score of zero might not have a protective effect against CV events, stroke, and CV death. In our subgroup analysis of patients with intermediate- or high-risk, a CAC score of zero did not lead to downward risk reclassification for the incidence of major CV events. These patients still had a certain risk for MACEs, even after adjusting for other risk factors. These findings are supported by some studies that show one-fourth to one-third of total incident CVD events occur in individuals with a CAC score of zero (39), and that a significant proportion of patients with a CAC score of zero have CAD (40).

There are several possible explanations for this discrepancy in results: (1) patients who had a CAC score of zero could have an atheroma burden, including significant stenosis, with no coronary calcification (41) [non-calcified plaque is more prevalent in Asians, especially among young smokers (42)]; (2) Agatston's technique may fail to detect very small or less dense calcifications that are more likely to cause acute coronary syndrome or MI than are densely calcified plaque (43, 44) [altering the Agatston criteria by eliminating size and reducing HU thresholds to ≥120 HU can improve detection of subtle calcification (45)]; (3) there could be disease plaque progression, expecially in young individuals [a study by Lee et al. from the Korea Initiatives on Coronary Artery Calcification (KOICA) registry (46) found that among 6,268 participants, 11.5% experienced CAC progression during follow-up (median, 109 months), even among those with a CAC score of zero]. Nevertheless, the power of a zero CAC score can be a strong downward risk classifier in older patients whose CAD burden predominantly involves calcified plaque (47). However, in patients under 40 years of age, a CAC score of zero does not reliably exclude obstructive CAD since they tend to have a higher burden of non-calcified plaques (48). Thus it is important to emphasize that CAC scores should be interpreted along with clinical risk factors, not alone. Currently, the AHA/American College of Physician (ACP) recommendation in 2018 (49) suggests that adults aged 40–75 years without DM, and with LDL-C levels ≥70–189 mg/dl, having a 10-year ASCVD risk score of ≥7.5%–9.9%, may delay statin therapy if the CAC score is zero. Exceptions to this are cigarette smokers, those with DM, and those with a strong family history of premature ASCVD.

Although CAC scanning showed significant promise in improving assessment of CV risk, the optimal method of integrating CAC data with other risk factor data is unclear. Currently, a common approach to incorporating CAC results is to use a CAC score of 100 Agatston units as a cut-off to downgrade or upgrade CV risk. While such a cut-off would provide a clinically simple approach when combined with risk factors, it overlooks the spectrum of risk along gradations of both CAC scores and risk factor levels. In fact, even among patients with a CAC score of zero, CV risk varies widely depending on the severity of other risk factors (13). An alternative approach is to integrate actual scores and risk factor data into a combined risk calculator to provide a more actuarial assessement of CV risk.

To support this idea, we modeled use of CAC scores as ln(CAC + 1) and demonstrated the efficacy of stratifying the Thai CV risk score in terms of the NRI index. In our study, we found that adding ln(CAC + 1) to the Thai CV risk score improved the C statistic from 0.703 to 0.716 and resulted in an NRI index of 0.06 (0.02, 0.10). The addition of the CAC score was associated with an increased number of patients who were correctly reclassified from high to low risk, although it also increased the (larger) number of patients who were incorrectly reclassified from low to high risk. A systemic review by Lin et al. (8) reported that adding the CAC score to traditional CVD risk models was associated with an improvement in model discrimination, with a pooled gain in the C statistic ranging from 0.018 to 0.144, and an NRI ranging from 0.084 to 0.35. Using these results as a benchmark, the incorporation of CAC score into the current Thai CV risk score showed a further modest improvement in the NRI index.

Seeking greater accuracy of risk prediction, we developed a new pooled cohort equation based on data from our cohort. We demonstrated that age, gender (male), hypertension, DM, dyslipidemia, hyperuricemia, arterial stiffness index, and the presence of coronary calcium [ln(CAC + 1)] were significant predictors. Currently, there is no integrated CAC and risk factor calculator available to determine the risk of ASCVD specifically for Asian populations. Using these parameters including CAC score for a new pooled cohort risk model, the C statistic further increased from 0.716 to 0.771. When compared to the results of the MESA (9), and Astro-CHARM (50) investigators, who developed integrated CAC calculators to predict CHD risk and 10-year risk of ASCVD, respectively, inclusion of a CAC score into the MESA risk score calculator offered significant improvement in risk prediction (C-statistic 0.80 vs. 0.75, *p* < 0.0001). Similarly with the Astro-CHARM, addition of a CAC score significantly improved risk prediction (C-statistic 0.817 vs. 0.784, *p* < 0.0001).

With our revised prediction model, in addition to incorporating the degree of coronary calcium, our study stands out for its integration of several novel risk predictors. These include the arterial stiffness index measured by CAVI and hyperuricemia. This comprehensive approach covers a broader spectrum of factors that contribute to advancing research in ASCVD risk prediction, thereby improving the precision of our predictions. To the best of our knowledge, there is no widely utilized ASCVD risk score that includes these risk factors as components of the risk assessement.

CAVI has gained popularity as an adjunctive tool for detecting early physiologic changes associated with subclinical atherosclerosis (51, 52). In our previous study (23), we found that a CAVI value of ≥9 improves the prediction of future MACEs and serves as an additional predictor, particularly among patients with noncalcified plaques or CAC scores <100. The arterial stiffness index offers a noninvasive and simple test that is associated with future CV events and mortality (53). However, it is based mainly on data which originate from Asian populations since it is not widely used in Western countries, and it is not recommended in any guidelines. In the future, as more evidence accumulates, CAVI may prove to be a valuable and widely accepted adjunctive tool to integrate into risk prediction models.

The link between hyperuricemia and the risks of gout, hypertension, and CKD is well-established (54, 55). Elevated serum uric acid can lead to endothelial dysfunction through inflammation and oxidative stress, as well as the formation of unstable lipid plaque in coronary arteries and other vascular territories, ultimately resulting in atherosclerosis (56, 57). Our study is the first to demonstrate the benefit of integrating hyperuricemia into the risk model, enhancing its ability to predict occurrence of CV diseases. In contrast, CKD, a recognized risk-enhancing factor for CV diseases (58), did not reach statistical significance as a risk predictor in the revised model. This was likely due to an exclusion criterium of the coronary CT study proptocol, which enrolled only patients with serum creatinine levels <1.5 mg/dl. Consequently, our study lacked the full spectrum of CKD in its study population. Moreover, there are some discrepancies in the risk factors between our model and other risk prediction models, such as family history of CAD and smoking. These differences could potentially be explained by ethnic and genetic variations, and the higher representation of females in our cohort. To futher improve the accuracy of our risk calculation, it may be important to consider all possible risk-enhancing factors and specific lipid/biomarkers [such as lipoprotein (a), apolipoprotein B, hs-CRP] (59). In the near future, comprehensive artificial intelligence (AI) may contribute to the accuracy and precision of clinical and CAD assessments, improving the prediction of long-term ASCVD risk (60).

In summary, the addition of CAC appears to provide additional discrimination to traditional CVD risk assessment. However, the modest gain needs to be weighed against the costs, radiation risks, and potential cascades of downstream testing and revascularization procedures. Importantly, while CAC may influence patients to improve medical adherence and maintain healthy lifestyles, incorporation of CAC scores into traditional models has not been proven to reduce the risk of coronary or CVD events. Futher research is needed to assess the clinical utility and cost-effectiveness of this additional accuracy.

## Limitations of the study

The present study has several limitations that should be acknowledged. First, it was an observational retrospective cohort study and thus had inherent limitations. The available data may not have included all the relevant variables needed to adjust for potential confounding factors, including concurrent treatment. This could introduce bias in estimating the true effect of the CAC score to ASCVD risk prediction. Second, selection bias was a concern, since our study population was not fully representative of the general Thai population. The majority of our subjects were female and only a small proportion were classified as high-risk. Third, there was a potential for measurement bias, as the accuracy of CAC score measurement can vary across different imaging modalities, scanners and reading centers. Fourth, the study design limited the ability to establish causality. While this study provided valuable insignts into the association between the CAC score and ASCVD risk, it cannot demonstrate whether incorporating the CAC score into the ASCVD risk score will actually lead to improved prediction of clinical outcomes. Future prospective studies of Asian populations are necessary to confirm that addition of CAC scores do indeed refine their ASCVD risk scores and thereby provide clinical benefit. Last, it is essential to note that our cohort was not ethnically diverse, as all participants were Asian. Therefore, any observed disparities between this and other studies may, in part, be attributed to ethnic differences. But for this very reason, this study was done to address the lack of research data from this major global population.

## Conclusions

The use of CAC scores along with traditional risk factors resulted in a modest improvement in the predictive accuracy of Thai CV risk scores among asymptomatic patients. This was particularly true in the low- and intermediate-risk groups and resulted in a number of reclassifications. The incorporation of CAC into a new pooled cohort equation appeared to enhance the discrimination function of risk prediction. It is crucial to note that CAC serves as a modifier of risk assessment rather than as a stand-alone test. Its use should be integrated with consideration of other risk factors and experienced clinical judgement. Further research is necessary to determine whether a greater accuracy of ASCVD risk leads to improved disease management and in reducing the frequency of CV events.

## Data Availability

The raw data supporting the conclusions of this article will be made available by the authors, without undue reservation.
