# Supplementary material for: Utility of coronary artery calcium in refining 10-year ASCVD risk prediction using a Thai CV risk score
Source: Front Cardiovasc Med. 2023 Nov 2;10:1264640. doi: 10.3389/fcvm.2023.1264640 (PMC10652894; doi:10.3389/fcvm.2023.1264640)
Supplement: Supplementary file 1 [file Datasheet1.docx]

**Supplementary Table S1.** Baseline characteristics of study participants stratified by Thai CV risk score.

| Baseline Characteristic | Low-risk  (<10%) n=5243 | Intermediate-risk (10-<20%) n=1427 | High-risk  (>=20%) n=294 |
| --- | --- | --- | --- |
| CAC score level, n (%) |  |  |  |
| 0 | 3037 (57.9) | 370 (25.9) | 51 (17.4) |
| 1-99 | 1586 (30.3) | 551 (38.6) | 94 (32.0) |
| >100 | 620 (11.8) | 506 (35.5) | 149 (50.7) |
| Ln(CAC +1), median (range) | 0.0 (0.0, 8.2) | 3.7 (0.0, 8.1) | 4.7 (0.0, 8.5) |
| CAVI |  |  |  |
| ≥ 9 | 1453 (28.1) | 855 (60.6) | 223 (77.2) |
| < 9 | 3726 (71.9) | 555 (39.4) | 66 (22.8) |
| Age, years, mean (SD) | 56.55 (7.1) | 66.22 (5.9) | 73.04 (5.7) |
| Sex, n (%) |  |  |  |
| Male | 1625 (31.0) | 714 (50.0) | 175 (59.5) |
| Female | 3618 (69.0) | 713 (50.0) | 119 (40.5) |
| BMI, kg/m^2^, mean (SD) | 24.70 (3.6) | 25.53 (3.6) | 25.35 (3.9) |
| DM, n (%) |  |  |  |
| Yes | 855 (16.3) | 734 (51.4) | 203 (69.1) |
| No | 4388 (83.7) | 693 (48.6) | 91 (31.0) |
| Hypertension, n (%) |  |  |  |
| Yes | 2942 (56.1) | 1247 (87.4) | 274 (93.2) |
| No | 2299 (43.9) | 180 (12.6) | 20 (6.8) |
| SBP, mmHg, mean (SD) | 127.12 (15.7) | 142.65 (15.8) | 155.55 (20.2) |
| Dyslipidemia, n (%) |  |  |  |
| Yes | 3041 (58.0) | 1011 (70.9) | 206 (70.1) |
| No | 2200 (42.0) | 416 (29.2) | 88 (29.9) |
| Current/ex-Smoking, n (%) |  |  |  |
| Yes | 573 (10.9) | 340 (23.8) | 109 (37.1) |
| No | 4670 (89.1) | 1087 (76.2) | 185 (62.9) |
| CKD, n (%) |  |  |  |
| Yes | 176 (3.4) | 163 (11.4) | 91 (31.0) |
| No | 5066 (96.6) | 1264 (88.6) | 203 (69.1) |
| Uric acid >7 mg/dL |  |  |  |
| Yes | 582 (11.2) | 262 (18.4) | 77 (26.3) |
| No | 4623 (88.8) | 1161 (81.6) | 216 (73.7) |
| Family history of CAD, n (%) |  |  |  |
| Yes | 1890 (36.1) | 385 (27.0) | 72 (24.5) |
| No | 3353 (63.6) | 1042 (73.0) | 222 (75.5) |
| TC, mg/dL, mean (SD) | 211.02 (40.6) | 201.41 (40.5) | 193.86 (46.0) |
| HDL-C, mg/dL, mean (SD) | 54.54 (14.0) | 47.86 (11.6) | 44.34 (10.8) |
| LDL-C, mg/dL, mean (SD) | 133.66 (37.3) | 127.28 (36.8) | 123.46 (42.7) |
| Statins, n (%) |  |  |  |
| Yes | 3396 (64.8) | 1127 (79.0) | 230 (78.2) |
| No | 1847 (35.2) | 300 (21.0) | 64 (21.8) |
| Anti-hypertensive agents, n (%) |  |  |  |
| Yes | 2841 (54.2) | 1137 (79.7) | 250 (85.0) |
| No | 2402 (45.8) | 290 (20.3) | 44 (15.0) |

BMI, body mass index; CAD, coronary artery disease; CAVI, cardio-ankle vascular index; CKD, chronic kidney disease (eGFR < 60 ml/min/1.73 ml^2^); DM, diabetes mellitus; HDL-C, high-density lipoprotein cholesterol; LDL-C, low density lipoprotein cholesterol; SBP = systolic blood pressure; TC = total cholesterol

**Supplementary Table S2.** AIC and BIC of the Cox proportional hazard models based on inclusion of categorical CAC and ln(CAC+1) scores.

|  | AIC | BIC |
| --- | --- | --- |
| CAC score category | 9404.3 | 9418.1 |
| ln(CAC +1) | 9398.3 | 9405.1 |

AIC, Akaike Information Criterion; BIC, Baysian Information Criteria
